# Eating Disorders and Addictive Behaviors: Implications for Human Health

**DOI:** 10.3390/nu15173718

**Published:** 2023-08-25

**Authors:** Fernando Fernández-Aranda, Roser Granero, Susana Jiménez-Murcia

**Affiliations:** 1 CIBER Physiology of Obesity and Nutrition (CIBEROBN), Carlos III Health Institute, 28029 Madrid, Spain; roser.granero@uab.cat (R.G.); sjimenez@bellvitgehospital.cat (S.J.-M.); 2 Psychoneurobiology of Eating and Addictive Behaviors Group, Neurosciences Programme, Bellvitge Biomedical Research Institute (IDIBELL), 08908 Barcelona, Spain; 3 Clinical Psychology Unit, University Hospital of Bellvitge, 08907 Barcelona, Spain; 4 Department of Clinical Sciences, School of Medicine and Health Sciences, University of Barcelona, 08907 L’Hospitalet de Llobregat, Spain; 5 Department of Psychobiology and Methodology, Autonomous University of Barcelona, 08193 Barcelona, Spain

## 1. Introduction

Eating disorders (EDs) are mental health diseases characterized by dysfunctional eating patterns, including restrictive eating, avoidance of foods, binge eating, and compensative behaviors to avoid weight increases and promote thinness (purging, vomiting, laxative/diuretics misuse, and compulsive exercise). These eating-related behaviors occur with concurrent severe negative consequences which affect physical, psychological, and social function [1]. As a consequence, the onset and progression of EDs often lead to comorbidity with other multiple psychiatric disorders, disabilities, and mortality rates [2].

There are a diverse range of ED types, with the most common being anorexia nervosa, bulimia nervosa, binge-eating disorder, and other specified feeding and eating disorders [3]. Overall, lifetime EDs have been identified in around 5% of the general population among developed societies [4]. EDs are now on the rise worldwide, and the large increases in estimated prevalence during recent decades (rates more than doubled between 2000 and 2020 among all people) have pointed to the need for new studies assessing the etiology and underlying mechanisms of these complex eating-related problems among clinical and population-based samples [5].

Behavioral addictions (BAs) are non-substance-related addictions characterized by an incapacity to resist impulses toward rewarding stimuli despite the adverse consequences. Aside from gambling and gaming disorder (the two most frequent conditions within the spectrum of behavioral addictions), other maladaptive and uncontrolled behaviors include compulsive sexual behaviors and compulsive buying, among other clinical conditions. The estimated prevalence differs depending on the BA subtype, geographical area, and measurement tools, with values ranging between 0.1% and 6.0% for gambling-related problems [6], 3% and 6% for gaming disorder [7,8], and 5% for compulsive buying [9]. The high prevalence of BAs over the last two decades has attracted increased scientific interest, mostly towards those related to the use of technology [10].

This Special Issue aims to identify the underlying triggers of EDs and BAs, two complex conditions with different diagnostic criteria but that exhibit common clinical features and functional processes. In the following sections, we present the studies and contents covered in this topic, which provide a new empirical basis for developing reliable assessment tools and evidence-based intervention plans (tailored to the individual needs of patients) from a multidisciplinary perspective.

## 2. Young People: A High-Vulnerability Group for EDs

Etiological research has identified multiple risk factors as predictors of ED onset and progression, with age being one of the variables receiving special attention. The stage between adolescence and early adulthood has been described as a typical age of onset for EDs since this is a crucial period for significant changes [11]. At the biological level, significant changes are observed in morphological, physiological, hormonal, and cognitive functions. At the psychological level, this is a key period of emotional adjustment aimed at facilitating the formation of an identity. At the social level, young people typically challenge parental authority. They make great efforts to be accepted in certain social groups. During this complex process, increased self-awareness contributes to dysfunctional self-doubts and unfavorable social comparisons, which are also highly impacted by sociocultural pressures related to self-image and lifestyle habits (resulting in body dissatisfaction, dieting, and other compensatory behaviors). Individuals with high emotional hyperreactivity and low executive control of their own behaviors can exhibit dysfunctional behavioral patterns that can be conceptualized as precursors of behavioral problems and psychopathology. Finally, young people represent a vulnerable group for developing and progressing mental illness.

Five manuscripts in this Special Issue analyzed data collected among children and adolescents. First, Rios et al. explored the relationships between food addiction and dietary restraint within a sample of adolescents between 13 and 16 years of age in a longitudinal study over a 2-year period of follow-up [12]. A cross-lagged panel revealed that early food addiction more strongly predicted future dietary restraint than the inverse relationship. The authors concluded that their results validated models of addiction among adolescents, particularly those sustaining that restraining behavior constitutes a way to control addictive behaviors. Consequently, strategic plans specifically aiming to ameliorate food addiction (such as nutritional-health-promoting school and home settings) may be crucial during adolescence to prevent later dietary restraint behaviors and the onset of EDs.

Second, the study by Via and Contreras-Rodríguez was a narrative review of the brain basis of binge-eating disorder among children and adolescents, a critical period for neurodevelopment [13]. The review’s scope points special attention to the deficits in the emotional regulatory mechanisms identified in this disorder, mostly involving reward-based processing and inhibitory mechanisms related to self-regulation. Similar findings in adult studies, neurocognitive tests, and MRI tasks among youths suggest that hypoactivation in inhibitory control circuits and the hyperactivation of hug regions of reward systems are signals of binge-eating patterns. The study also suggests that ultraprocessed food and drink exposure during childhood may promote changes in the frontolimbic brain circuits, which then contribute to dysfunctional emotional regulation processes during adolescence.

Continuing with the study of youth, another work in this Special Issue, conducted by Matusik and colleagues, also suggests that within this period, specific groups that a priori could be considered as low-vulnerability can, in turn, trigger a nascent obsession with food, eating styles, and body image. The authors estimated a high prevalence of subjects with a positive score in screening tests for EDs among university dietetics students (close to 46%) [14]. Although it may seem inconsistent, these (young) students, who receive extensive education on proper nutrition and healthy, fashionable eating styles, could also develop abnormal relationships with food, and the authors suggest that this is probably due to their exacerbated desire to become perfect role models in eating and body appearance. These individuals are also often prone to stressful events including moving to college, making decisions about their future, or simply taking exams. The authors also observed that around 14% of nutrition students were in the range of overweight to obese at the start of university, and this evidence suggests that ED problems could be underlying in these concrete individuals prior to the beginning of their studies (individuals may view their studies as a way to deal with their inappropriate eating styles, but in the end, they increase their preoccupation with healthy eating).

Similar results were observed in the fourth study of this issue analyzing data among a young population. Grajek et al. compared university students distinguished by their fields of study: health-related areas versus non-health-related areas [15]. They observed a high prevalence of young individuals reporting behaviors characterized by a lack of control over intake (20.7%) and emotional eating (37.9%), independent of their course. This study concluded that among university students, those with lifestyles characterized by inappropriate diets, low rates of physical activity, and high levels of perceived stress are likely to develop unhealthy eating patterns independent of their chosen subject. Ultimately, arrival at university is one of the most important changes that take place in the lives of young people. This period of liberation is usually accompanied by an increase in stress levels, and students’ initially high expectations of study motivation can be hampered because they find themselves experiencing concurrent changes that overall affect their lifestyle.

This Special Issue also includes a multicenter, cross-sectional study aimed at exploring behaviors, attitudes, perceptions, and barriers to engagement among children and adolescents (12 to 17 years old) with obesity, caregivers, and healthcare professionals [16]. The results showed that around one-quarter of children with obesity and around half of caregivers perceived that the child’s weight was in the normal range; additionally, almost 95% of caregivers perceived the child in their care to be in good health. These results evidence the tendency for parents/caregivers to misperceive their child’s weight (they underestimate overweight status), and therefore, their severe inability to recognize obesity as a disease during this stage. Given that many caregivers in the study (around 40%) also felt that possibly being overweight was not a relevant problem because the children would get thinner as they grew up, this will lead to a denial of the problem and delay early intervention. The authors recommend improved communication systems between all the individuals involved in the process (children and adolescents living with obesity, caregivers, and healthcare professionals), an adequate identification of the multiple barriers to addressing weight-related problems, and improved health education on nutrition and its correlates.

## 3. Other Risks for EDs (Affecting Any Age Group)

The prevalence of EDs has increased across all social sectors in developed countries. In addition to the risks that are typically present at different points in the life course, epidemiological research has identified additional threats to EDs that can have an impact at any age/stage of life, depending on the broader sociocultural and psychological context. For example, participating in competitive sports may increase the chances of developing ED outcomes [17]. Professional athletes focus most of their lives on sports and they can relate their athletic performance to restricted dietary and dysfunctional nutritional intake. These individuals can also emphasize appearance and overvalue the belief that lower body weight will improve performance [18]. The study by Ibañez-Caparrós and colleagues included in this issue observed that, when comparing ED patients who were professional athletes with those who were not, the athletes showed less body dissatisfaction and better psychological performance [19]. However, within the athlete group, individual sport activity and aesthetic sports (such as gymnastics, diving, and figure skating) were associated with worse clinical profiles (higher eating-disorder symptom levels and more comorbid psychological problems) and poorer therapy outcomes. The authors outlined the need for adequate prevention plans for sports organizations and professionals to support athletes. Special attention must be paid to aesthetic sports, which involve judging individual/team performance based on a complex set of rules including appearance. Weight-dependent sports (which divide athletes into weight classes) also deserve special attention, since athletes could normalize the use of compensatory behaviors like vomiting, laxatives, diuretics, and even dehydration as weight control mechanisms.

Stress can impact eating patterns and trigger EDs. Concretely, stressful events occurring in the social and family domains have been identified as powerful risk factors for the onset and evolution of eating-related problems. And, simultaneously, studies have observed that EDs also greatly impact family and social functioning. In this Special Issue, the study carried out by Momeñe and colleagues used path analysis to analyze the underlying relationships between dysfunctional features strongly related to ED profiles (the perception and fear of loneliness, inadequate coping mechanisms to regain control over stressful events, and social isolation) and the likelihood of suffering intimate partner violence (IPV) throughout life [20]. Among a population-based sample composed of young participants, it was observed that specific ED symptoms were directly related to IPV (high drive for thinness, ineffectiveness, perfectionism, interoceptive awareness, impulsiveness, and social insecurity). The study also showed that fear of loneliness was a mediating link between ED symptoms and received violence, but specifically among the high social withdrawal stratum. The authors concluded that the results were consistent with a bidirectional model between EDs and IPV, with central features such as fear of loneliness and social withdrawal acting as mediating links. In this sense, EDs may be interpreted as the consequence of employing dysfunctional mechanisms for coping with the highly adverse consequences of received violence. In addition, with the progression of the disorder, the severity of eating symptoms and other individual characteristics (such as fear of loneliness and social withdrawal) contribute to reinforcing the likelihood of establishing violent partner relationships.

The study by Hoover and colleagues was also focused on how individuals cope with stress levels as a potential risk for ED-related problems [21]. Using a mediational model, these authors tested the relationships between elevated perceived vulnerability to disease and increased fear of fat and cognitive restraint (defined as the control over food intake with the aim of regulating body weight) and compensatory behaviors. Among a sample of *n* = 247 adults (men and women aged from 21 to 70 years), it was found that perceived infectability and germ aversion directly predicted compensatory behaviors, while fear of obesity partially mediated the association. In addition, the participants’ sex was not identified as a moderator variable, indicating invariance in the structural coefficients for men and women. This suggested that reducing fear of fat among individuals who experience high perceived vulnerability to infection and disease may be a way to reduce disordered eating (e.g., delivering interventions and cognitive–behavioral techniques to cope with internalized weight stigma).

Finally, persons exposed to comorbid mental conditions represent a further group at significantly elevated risk of EDs. The presence of psychological problems is associated with a set of risks and vulnerabilities, and therefore, the onset and progression of a mental condition raise the odds of concurrent psychiatric diseases. A range of factors have been identified for the comorbid presence of mental disorders, including elevated rates of substance consumption to cope with distress and negative mood states, an unhealthy diet, and unhealthy lifestyles (diminished physical activity and disturbed sleep patterns). Current systematic reviews confirm that, for some individuals, EDs may be chronic disorders that persist from childhood to adulthood, and that patients with EDs are at a higher risk of developing multiple comorbid mental clinical states [22]. The study carried out by Miranda-Olivos and coworkers, included in this Special Issue, assessed how the presence of EDs with comorbid addictions (food addiction and/or substance use) impacts clinical profiles and treatment outcomes [23]. The results showed that the presence of addictive behaviors at baseline was associated with a higher risk of dropout during therapy, and that within the patients with poor treatment outcomes, a comorbid ED plus at least one addiction-related behavior was linked to a clinical profile characterized by greater ED symptom severity, a worse psychopathological state, and more dysfunctional personality traits. The authors concluded that while the presence of addictive behaviors could show a low direct impact on treatment efficacy among ED patients, these comorbid conditions could exert an indirect effect on interventions acting as mediating variables, contributing to worsening clinical profiles at baseline (global distress and ED severity).

## 4. The Biology of EDs and BAs: Genetic and Neuropsychological Markers

Evidence suggests the existence of multiple biological markers related to the onset and progression of EDs and BAs, including genetic and neuropsychological processes. The study by Solé-Morata et al. [24], included in this issue, tested the underlying mechanisms contributing to BA severity through path analysis, specifically among patients seeking treatment for gambling disorder. Overall, 183 nucleotide polymorphisms (SNPs) of several neurotrophic factors (NFTs) were genotyped, and 4 were selected and analyzed based on the results obtained in previous research: (1) rs796189, the presence of genotypes “AG/GG” (dominant model) and “AG” (overdominant model); (2) rs3763614, the presence of genotypes “CC” (codominant and dominant models) and “CC/TT” (overdominant model); (3) rs11140783, the presence of genotype “CC” (codominant model); and (4) rs3739570, the presence of genotypes “CC” (dominant model) and “CC/TT” (overdominant model). The results showed a complex vulnerability model including the direct and indirect impacts of both the genotype (single SNPs but also haplotype blocks) and phenotype (sociodemographic, psychosocial, and clinical factors) on the severity of gambling symptoms, which is consistent with etiological models of this disorder that include genetic and environmental factors [25].

The etiology of gambling disorder also identified neurocognition as a key domain in characterizing and maintaining the disease. Overall, BAs are associated with a distinct pattern of neurocognitive functioning differentiated by impaired top–down executive control and bias risk–reward processing. Theoretical models of addiction propose a hyperactive drive/reward salience network (a particular affectation was observed in the orbitofrontal cortex, striatum, and dorsal anterior cingulate cortex), in parallel with reduced executive functioning and cognitive control over behavior (related to decreased activity in the inferior frontal cortex and ventral anterior cingulate cortex) [26]. As a consequence, behavioral disinhibition and reward-driven decision making are observed in treatment-seeking patients at baseline (the dysfunctional level is interpreted as a measure of the addictive severity), and are also predictive of treatment relapse [27]. The manuscript by Mestre-Bach and Potenza [28] included in this issue is a state-of-the-art review about specific changes in the brain as a consequence of interventions in both BA- and ED-related conditions (specifically food addiction and binge eating). Specific attention was paid to ventral striatal activation and the related circuitry as a biomarker for these clinical conditions and their recovery, due to their role in reward processing systems. One main implication of the research is that neuropsychological performance should be considered in early detection plans, as well as potentially viable targets for designing novel, more effective interventions (pharmacological, psychobehavioral, and neuromodulatory), aimed towards the activation of specific brain areas involved in reward processing and its connectivity with others.

Turning to the field of EDs, one key domain in their etiology and development is impulsivity [29,30]. This is a complex transdiagnostic construct implied in multiple separate neuropsychological and behavioral dimensions [31] and strongly related to inhibitory control responses and cognitive decision-making processes [32,33]. Impulsivity has also proved to impact therapy response in both short- and long-term outcomes. The study by Testa and colleagues carried out in a sample of *n* = 37 female ED patients using cognitive behavioral therapy (a treatment recommended by most evidence-based guidelines) [34] found that poor inhibitory control at baseline (measured in a Stroop task) was a predictor of poor remission of ED symptom levels at the end of the therapy, while higher novelty seeking (a personality trait defined as the preference for new experiences with intense emotional sensations and risk taking) and poorer inhibition in event-related potentials registered in an emotional go/no-go task negatively impacted symptomatology remission at 2-year follow-up after the treatment [35]. This empirical evidence is consistent with other studies that showed associations between lower cognitive and behavioral control and poor remission (a high risk of dropout and suboptimal remission) in the short term and at follow-up in patients with EDs [36]. The empirical evidence obtained in the study by Testa et al. urges the need of the early detection of ED signals, including lack of inhibition, with the aim to plan precise intervention plans to improve treatment effectiveness. Therapeutic approaches with inhibitory control training with general or food-specific stimuli could be good candidates for ED patients with difficulties in inhibition [37,38,39].

## 5. Signals Regulating Energy Homeostasis among EDs and BAs

The homeostatic system is implied in control of feeding based on the regulation of the energy balance (motivation to eat is increased following a reduction in energy stores). Contrarily, the hedonic system implied in feeding is based on the reward-based regulation system and can override homeostatic signals during periods of energy abundance through a rise in the desire for highly palatable foods. Studies have analyzed the control of energy homeostasis and the pathogenesis of EDs, but new empirical evidence is required to further understand energy balance.

In this vein, the study by Nieto et al. [40] included in this issue analyzed the association between the digestive process that follows meal ingestion and a postprandial experience that involves homeostatic sensations (satiety and fullness) with a hedonic dimension (digestive well-being and mood). Based on the Pavlovian conditioning model and the hypothesis that the postprandial experience depends on the characteristics of the individual (intestinal sensitivity, digestive function, and other cognitive/emotive factors) and the meal (organoleptic and the amount and composition), the authors observed that pairing a pleasant meal with an experimentally induced aversive sensation conditions the postprandial response to the subsequent consumption of the same meal. The study also observed that aversive conditioning did not contribute to a homeostatic sensation and physiological digestive response, but significatively impaired the hedonic practice. As a consequence, it was suggested that aversive postprandial conditioning may be considered for the treatment of diverse health conditions, such as obesity, hypercholesterolemia, diabetes, and functional gut disorders. Also, the reinforcement of postprandial rewards and food balance could help counteract natural neophobias of new/unknown foods (for example, in children or patients with autism spectrum disorders) and promote ingestion in individuals with nutritional deficits (for example, patients with anorexia as a consequence of oncological treatments).

Another study included in this Special Issue was also focused on the study of signals involved in energy homeostasis, specifically in patients who met diagnostic criteria for gambling disorder [41]. An analysis of diverse gut hormones (ghrelin (an appetite-stimulator) and liver-expressed antimicrobial peptide2 (LEAP2, an endogenous antagonist of growth hormone secretagogue receptor)) and adipocytokines (leptin and adiponectin) revealed that, compared with a healthy control group, patients with gambling problems presented increased plasma ghrelin and lower LEAP2 and adiponectin concentrations, while no differences in leptin levels were found. The authors concluded that endocrine functions are related to reward processing involved in substance- and non-substance-related addictive processes, and therefore, they may have therapeutic implications because of the relationship with intensifying craving and relapsing.
